# On the Treatment of Intractable Strictures of the Urethra by Internal Incisions

**Published:** 1861-01

**Authors:** 


ART. VIII.—*Du Traitement par L'Urêthrotomie Interne des Rétrécissiments Organiques de l'Urèthre, Réfractaires aux Moyens Ordinaires. Thèse pour Le Doctorat en Médecine.* Par FELIX-EUGENE DUPIERRIS, M.D. 4to., pp. 78. Paris: Rignoux, 1860.

*On the Treatment of Intractable Strictures of the Urethra by Internal Incisions. Thesis for the Degree of Doctor of Medicine.* By FELIX-EUGENE DUPIERRIS, M.D.

IN this inaugural thesis, presented to the Academy of Medicine of Paris, and published by its authority, Dr. Dupierris has succeeded in collecting from every quarter a sufficient amount of information on the treatment of obstinate urethral strictures by urethrotomy, to form a very creditable brochure. After having treated of the causes, pathological anatomy, situation, structure, and different varieties of intractable strictures, the author occupies the greater portion of the work with the consideration of internal division, not neglecting to mention the methods of treatment by simple and forced dilatation, cauterization, excision, and by the perineal section.

The brochure gives promise of early usefulness and distinction; and we trust that the son may earn for himself a reputation worthy that of his father, Dr. Martial Dupierris, one of our most valued collaborators. We hope the day is not distant when students of this country will emulate the ambition of their Parisian brethren, in producing inaugural dissertations, alike creditable to themselves and to their teachers.

